# The implications of various gene variant combinations on breeding values for Awassi ewe milk production

**DOI:** 10.14202/vetworld.2023.2411-2415

**Published:** 2023-12-05

**Authors:** Khaleel I. Jawasreh, Ahmad H. Al-Amareen

**Affiliations:** 1 Department of Animal Production, Faculty of Agriculture, Jordan University of Science and Technology, Box 3030, Irbid 22110, Jordan; 2 Livestock Directorate, National Agriculture Research Center, Albaqa’a 19381, Jordan.

**Keywords:** Awassi sheep, breeding value, milk production

## Abstract

**Background and Aim:**

Milk production is an important factor to consider in selecting Awassi sheep. This trait is influenced by various genes that can be managed to boost production. The breeding values (BVs) for milk production in Jordan’s Awassi sheep flocks have been established. This study determined how combined gene variants of *BLG*, *PRL*, *CSN3*, *CSN1S1*, and *CSN2* affect the estimated BVs (EBVs) for milk production in Awassi sheep.

**Materials and Methods:**

Milk and blood samples were collected from 391 Awassi sheep, which was then subjected to molecular analysis through sequencing in order to identify potential alleles and genotypes that could be linked to the EBVs of milk.

**Results:**

The predicted BVs for milk were significantly influenced by *PRL* and *CSN3* gene variants. Through performing the act of epistasis, the interactions of *BLG* with *CSN3* and *–CSN1S1* greatly impacted EBVs for milk production. Likewise, the three-way interaction among *PRL*, *CSN3*, and *CSN1S1*, as well as the combined effect of *CSN3* with *CSN1S1* and *CNS1* significantly improved BVs for milk production. When the breeding selection program incorporates the polymorphisms of these genes, gains in milk production can be obtained.

**Conclusion:**

Alleles within the examined genomic areas are crucial for evaluating BVs and maximizing genetic gain in milk production.

## Introduction

In Jordan and the Middle East, Awassi sheep are highly valued for their high-quality milk and meat, as well as their relatively low survival and production requirements [1, 2]. Nearly 6 billion people consume milk and milk products for their high nutritional value [3]. The colloidal and suspended components of milk determine its quality. Many people prefer sheep’s milk due to its higher protein and fat contents compared with milk from other species [4]. Sheep’s milk is also a natural source of probiotics, and κ-casein (CSN3) has shown potential as a natural antioxidant [5]. However, using synthetic antioxidants has a level of toxicity that can be avoided by natural antioxidants such as α, β, and CSN3 caseins [6]. Instead of using antihypertensive medications with numerous adverse effects, β-casein has shown potential in blocking the angiotensin-converting enzyme responsible for raising blood pressure. Such inhibitory activities have resulted in a significant demand for β-casein due to their high safety profile [7]. Nutrition, weather, animal health, and genetic potential are just some variables affecting milk production and quality [8, 9]. Estimated breeding values (EBVs) for such traits are crucial because several genes influence milk and its components [10, 11]. The productivity of indigenous breeds should be increased through genetic and environmental methods to promote global food security. This endeavor would help emerging countries that are currently struggling to meet increasing milk demand due to poor milk production from their local breeds [12, 13].

Many genes affect milk production in sheep and play an important role in enhancing or diminishing productivity. *CSN1S1*, *CSN2*, *CSN3*, *BLG*, and *PRL* affect milk quality and quantity and are among the genes that can modify milk production features. For instance, *CSN3* is essential for the stability of casein, which is the most abundant milk protein [14, 15]. *BLG* is the most prevalent protein in whey and is polymorphic in sheep. Polymorphisms affect the components of sheep’s milk [14, 16]. *PRL* directly impacts the quantity of milk by inducing mammary epithelial cell (MEC) proliferation, which, in turn, increases milk productivity [17, 18]. However, selecting animals with higher milk yield is challenging, considering the different genes affecting milk production and their interactions [19, 20].Therefore, determining the relationship between EBVs and certain genes in sheep may significantly facilitate the selection process, avoiding the need to wait for generations of progeny before making selection decisions.

This study investigated the relationship between the EBVs for milk production in Awassi sheep and *BLG*, *PRL*, *CSN3*, *CSN1S1*, and *CSN2* variants, specifically the effects on the EBVs of the individual genes and their interactions.

## Materials and Methods

### Ethical approval

All experimental protocols involving animals were approved by the Animal Care and Use Committee, (approval Number 36/12/4/16) Jordan University and Science Technology.

### Sample collection and analysis

Full milk records and blood samples of 391 Awassi ewes were collected for analysis. The procedures, protocols, and field work location and period were as described by Jawasreh *et al*. [14].

### Estimation of BVs and amplification of genomic DNA

We performed quantitative genetic analysis to generate EBVs, as described previously by Jawasreh *et al*. [10], In a nutshell, the mixed model used in the ASreml program’s Individual Animal Model was used to estimate phenotypic and genetic parameters, including estimated breeding values (EBVs).We performed DNA extraction, amplification procedure sequencing, and mutation detection using the protocols proposed by Jawasreh *et al*. [14, 21].

### Statistical analysis

Statistical analysis was performed to analyze the effects of the genotypes identified on EBVs using SAS/STAT® software (version 9.1, SAS Institute Inc., Cary, NC, USA). We performed the general linear model procedure using the five genotypes and their possible interactions in the statistical model used.

## Results

### Estimated breeding values and gene combinations

**Figure-1:**
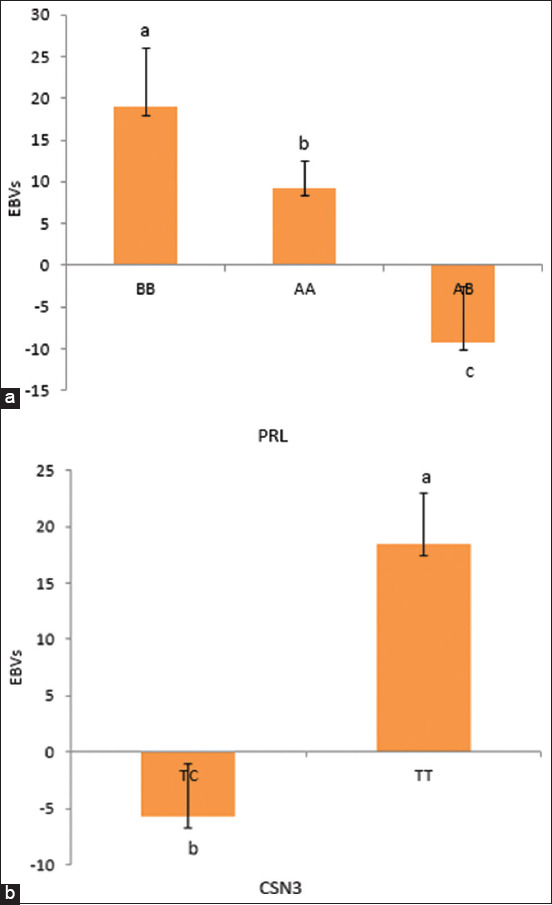
(a) Estimated breeding values for single effect of PRL on milk production in Awassi sheep. Vertical bars represent the mean ± SE of triplicates, and values with different superscripts are significantly different (p < 0.05). (b) Estimated breeding value for single effect of CSN3 on milk production in Awassi sheep. Vertical bars represent the mean ± SE of triplicates, and values with different superscripts are significantly different (p < 0.05). SE=Standard error.

**Figure-2:**
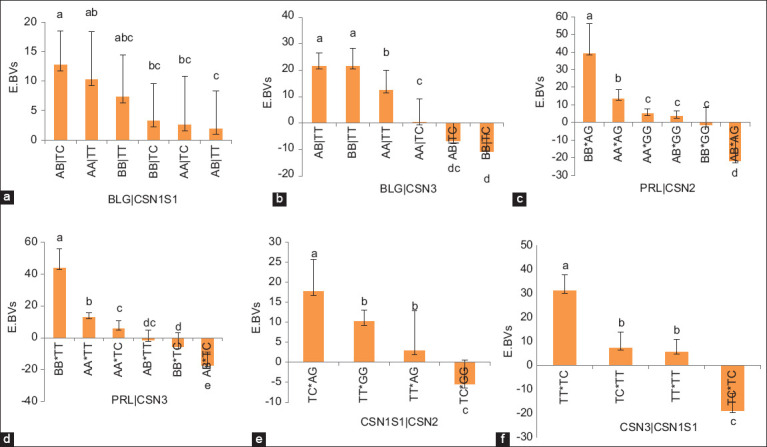
(a) Estimated breeding values for combined effect of BLG and CSN1S1 on milk production in Awassi sheep. Vertical bars represent the mean ± SE of triplicates, and values with different superscripts are significantly different (p < 0.05). (b) Estimated breeding value for combined effect of BLG and CSN3 on milk production in Awassi sheep. Vertical bars represent the mean ± SE of triplicates, and values with different superscripts are significantly different (p < 0.05). (c) Estimated breeding value for combined effect of PRL and CSN2 on milk production in Awassi sheep. Vertical bars represent the mean ± SE of triplicates, and values with different superscripts are significantly different (p < 0.05). (d) Estimated breeding value for combined effect of PRL and CSN3 on milk production in Awassi sheep. Vertical bars represent the mean ± SE of triplicates, and values with different superscripts are significantly different (p < 0.05). (e) Estimated breeding value for combined effect of CSN1S1 and CSN2 on milk production in Awassi sheep. Vertical bars represent the mean ± SE of triplicates, and values with different superscripts are significantly different (p < 0.05). (f) Estimated breeding value for combined effect of CSN3 and CSN1S1 on milk production in Awassi sheep. Vertical bars represent the mean ± SE of triplicates, and values with different superscripts are significantly different (p < 0.05). SE=Standard error.

**Figure-3:**
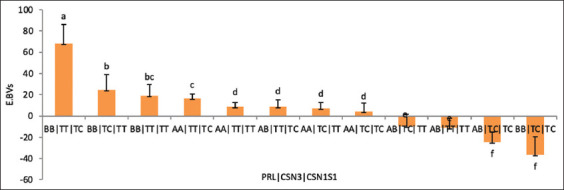
Estimated breeding values for combined effect of PRL, CSN3, and CSN1S1 on milk production in Awassi sheep. Vertical bars represent the mean ± SE of triplicates, and values with different superscripts are significantly different (p < 0.05). SE=Standard error.

The effects of individual and combined genes on EBVs for milk production in Awassi sheep are shown in Table-1. *PRL* and *CSN3* significantly affected the EBVs of milk production (p < 0.05). However, the interaction between the different genes *(BL*G*/SN3, BLG/CSN1S1, PRL/CSN2, PRL/CSN3, CSN3/CSN1S1*, and *CSN1S1/CNS2*) substantially altered the EBVs (p < 0.05). However, EBVs did not differ between the other combination pairs of genes (p > 0.05). The means and standard errors of the least squares for the effects of single and combined genotypes on EBVs for milk production were calculated. As indicated in Figure-1a, the *PRL* polymorphism, specifically, the BB genotype, had a significant effect on the EBVs for milk production (19.0 ± 7.0, p < 0.01), followed by the AA and AB genotypes (9.3 ± 3.1, -9.2 ± 6.6, respectively, p < 0.05). For *CSN3*, the TT genotype had the most significant impact on BV (Figure-1b), followed by the TC genotypes (p < 0.05). The results for the investigation on the combined influence of the *BLG*, *PRL*, *CSN3*, *CSN1S1*, and *CSN2* genotypes on EBVs for milk production are shown in Figures-2a–f. The interaction between *BLG* and *CSN1S1* had a significant impact on the EBVs, with the AB|TC genotypes resulting in the highest average EBVs (p < 0.05) compared with those of the BB genotype with the TC, AA|TC, and AB|TT interactions (Figure-2a). Furthermore, the interaction between the AB|TT and BB|TT genotypes of *BLG* and *CSN3* yielded the highest EBVs among all the gene combinations (Figure-2b). The most common genotype for the interaction between *PRL* and *CSN2* was BB|AG, with a least squares mean of 39.24 (p < 0.05) (Figure-2c). *PRL* × *CSN3* yielded the highest BVs (p < 0.05) among all combined genes, specifically the BB × TT genotype (Figure-2d). The TC|AG genotype from the interaction between *CSN1S1* and *CSN2* produced the highest EBVs (Figure-2e). The TT|TC genotype resulting from the combined group of *CSN*3 and *CSN1S*1 had the highest EBVs among all combined genes (Figure-2f). The EBVs for milk production for the interactions among *PRL*, *CSN3*, and *CSN1S1* and their genotypes are presented in Figure-3. The BB|TT|TC genotype showed the most significant effect on milk production EBVs above all genotypes (p < 0.01).

**Table-1:** p-values of the EBVs for milk production as affected by single and combined genes BLG, PRL, CSN3, CSN1S1, and CSN2 in Awassi sheep.

Gene	p-value of EBVs
*BLG*	0.954
*PRL*	0.0078
*CSN3*	<0.0001
*CSN1S1*	0.959
*CSN2*	0.388
*BLG × PRL*	0.547
*BLG × CSN3*	0.051
*BLG × CSN1S1*	0.035
*BLG × CSN2*	0.195
*PRL × CSN1S1*	0.852
*PRL × CSN2*	0.021
*PRL × CSN3*	0.041
*CSN3 × CSN1S1*	<0.0001
*CSN1S1 × CNS2*	0.006
*PRL × CSN3 × CSN1S1*	0.014

EBVs=Estimated breeding values

## Discussion

Milk production is a crucial criterion in the selection for dairy sheep. Milk production involves several genetic factors that can influence milk quantity and quality. The complexity of genotyping and defining an effective parameter can affect BVs for milk production in sheep [22]. Estimating BVs for Awassi sheep by acquiring their genetic profiles is considered highly effective [23]. To the best of our knowledge, this is the first investigation of *BLG*, *PRL*, *CSN3*, *CSN1S1*, and *CSN2* interactions in Awassi sheep. The phrase “difference in breeding values (EBVs) between genes” refers to the various ways in which genes might express themselves in relation to a breed and a variety of environmental circumstances [24].

Researchers worldwide have investigated different potential genes that substantially impact milk production in sheep [14, 19, 20, 21]. These genes include *CSN3*, *CSN1S1*, *CSN2*, *BLG*, and *PRL*. *CSN3* plays a critical role in micelle production and stabilization [25], which affects the production characteristics and digestibility of milk. However, the association between these markers and milk yield and composition in dairy sheep has not been thoroughly described in the literature.

As shown in Figure-1b, the *CSN3* genotypes had the biggest impact on the EBVs for milk production, with the TT genotype obtaining the highest EBVs. However, according to Jawasreh *et al*. [14] and Gras *et al*. [26], the *CSN3* genotypes have no appreciable impact on the quantity and quality of milk produced by the Awassi and Teleorman Black Head breeds. Thus, to increase milk production, assessing phenotypic data along with molecular data is crucial for modern breeding programs for dairy sheep [27].

Conversely, *PRL* directly affects milk production by stimulating the growth of MECs [28]. The current analysis on the impacts of the *PRL* genotypes on milk production EBVs revealed that the BB genotype yielded the highest EBVs (Figure-1a), followed by the AA and AB genotypes (p < 0.05). This outcome conflicts with several previously reported findings [14, 26, 27] that claimed significant superiority in milk output of the AA genotype. According to our investigation, the interactions of the *BLG*|*CSN3*, *BLG*|*CSN1S1*, *PRL*|*CSN2*, *PRL*|*CSN3*, *CSN2*|*CSN1S1*, and *PRL*|*CSN2*|*CSN1S1* genotypes significantly impacted the EBVs of milk production in Awassi sheep (Figures-2a-e and-3). Similarly, Jawasreh *et al*. [14] and Al-Amareen and Jawasreh [21] both reported a significant effect of the *BLG*|*PRL* and *CSN1S1*|*CSN2* genotypes on milk production in Awassi sheep.

The effects of combined genotypes on EBVs for milk production revealed in this study are an intriguing demonstration of the significant impact of genotype combinations and gene interactions on quantitative traits [29].

Identifying the genes that affect milk production provides a definite advantage in improving BVs for milk production. Furthermore, establishing the relationship between allelic variations and gene interactions can be a useful strategy for assessing BVs. Therefore, accurately determining genotype diversity can produce the greatest impact on BVs within a short generation period [30].

## Conclusion

The findings of this study demonstrate the importance of determining the impacts of *BLG*, *PRL*, *CSN3*, *CSN1S1*, and *CSN2* and their interactions on milk production EBVs. The interaction between *PRL* × *CSN3* × *CSN1S1* yielded the highest EBVs for milk production. Thus, considering these genes during breed selection signifies great potential for producing sheep with higher milk yields. Conversely, *BLG* × *CSN1S1* showed the lowest combined effect on milk production. Our study proves that gene variations and their interactions should be considered when creating selection programs for the quantitative genetic model used to determine BVs for significant gains in milk production.

## Authors’ Contributions

KIJ and AHA: Conceptualization and methodology, validation, formal analysis, investigation, resources, data collection, and drafted and revised the manuscript. Both authors have read, reviewed, and approved the final manuscript.
